# The Light-Induced WD40-Repeat Transcription Factor DcTTG1 Regulates Anthocyanin Biosynthesis in *Dendrobium candidum*

**DOI:** 10.3389/fpls.2021.633333

**Published:** 2021-03-17

**Authors:** Ning Jia, Jingjing Wang, Yajuan Wang, Wei Ye, Jiameng Liu, Jinlan Jiang, Jing Sun, Peipei Yan, Peiyu Wang, Fengzhong Wang, Bei Fan

**Affiliations:** ^1^Institute of Food Science and Technology, Chinese Academy of Agricultural Sciences, Beijing, China; ^2^Laboratory of Quality & Safety Risk Assessment on Agro-products Processing, Ministry of Agriculture and Rural Affairs, Beijing, China; ^3^Institute of Medicinal Plant Sciences, Sanming Academy of Agricultural Sciences, Sanming, China

**Keywords:** *Dendrobium candidum*, DcTTG1, anthocyanin biosynthesis, transcriptional regulation, transcription factor

## Abstract

*Dendrobium candidum* is used as a traditional Chinese medicine and as a raw material in functional foods. *D. candidum* stems are green or red, and red stems are richer in anthocyanins. Light is an important environmental factor that induces anthocyanin accumulation in *D. candidum*. However, the underlying molecular mechanisms have not been fully unraveled. In this study, we exposed *D. candidum* seedlings to two different light intensities and found that strong light increased the anthocyanin content and the expression of genes involved in anthocyanin biosynthesis. Through transcriptome profiling and expression analysis, we identified a WD40-repeat transcription factor, DcTTG1, whose expression is induced by light. Yeast one-hybrid assays showed that DcTTG1 binds to the promoters of *DcCHS2*, *DcCHI*, *DcF3H*, and *DcF3′H*, and a transient GUS activity assay indicated that DcTTG1 can induce their expression. In addition, DcTTG1 complemented the anthocyanin deficiency phenotype of the *Arabidopsis thaliana ttg1-13* mutant. Collectively, our results suggest that light promotes anthocyanin accumulation in *D. candidum* seedlings *via* the upregulation of DcTTG1, which induces anthocyanin synthesis-related gene expression.

## Introduction

*Dendrobium candidum*, a perennial herb of the orchid family, has been used for thousands of years in China as a traditional Chinese medicine and in functional foods (Ng et al., 2012), including teas, juices, wines, stews, and soups (Wei et al., 2016). *D. candidum* stems are green or red, with the latter having a higher anthocyanin content. Anthocyanins have a high nutritional value and are beneficial for human health (de Pascual-Teresa and Sanchez-Ballesta, 2008). Further, they have a broad spectrum of medicinal effects, including protection against liver injury, reduction of blood pressure, improvement of eyesight, and strong anti-inflammatory activity and anticancer properties (Konczak and Zhang, 2004). In addition, anthocyanins function to attract insect pollinators and protect plants against environmental stresses. However, the gene transcription regulatory mechanism underlying color formation in *D. candidum* stems has not been fully unraveled.

Anthocyanin biosynthesis is catalyzed by a series of enzymes, including chalcone isomerase (CHI), flavanone 3-hydroxylase (F3H), flavonoid 3′-hydroxylase (F3′H), flavonoid 3′,5′-hydroxylase (F3′5′H), dihydroflavonol 4-reductase (DFR), and anthocyanin synthase (ANS). These enzymes are encoded by structural genes, the expression of which is regulated by transcription factors (TFs; Broun, 2005). Key anthocyanin biosynthetic enzymes have been extensively studied (Zhang et al., 2014). Anthocyanin biosynthesis starts from the flavonoid synthesis pathway with the formation of chalcone from 4-coumaroyl CoA and malonyl CoA by chalcone synthase (CHS). Anthocyanidins undergo glucose-flavonoid 3-*O*-glucosyl transferase (UFGT) modification to increase their stability (Holton and Cornish, 1995; Zhang et al., 2014). Anthocyanin aglycones include delphinidin, cyanidin, pelargonidin, peonidin, malvidin, and petunidin (Cabrita et al., 2000).

Numerous TFs regulating plant anthocyanin biosynthesis have been identified to date. They include three main types: MYB TFs, basic helix-loop-helix (bHLH) factors, and WD40-repeat proteins. MYB-bHLH-WD40 (MBW) complexes play an important role in anthocyanin biosynthesis in various plant species (Xu et al., 2015). Environmental biotic and abiotic factors play important roles in plant secondary metabolism, including anthocyanin, flavonoid, and polysaccharide metabolism. In *Arabidopsis thaliana*, environmental stress factors, such as light, cold, and drought stresses and pathogen invasion, enhance anthocyanin accumulation (Chalker-Scott, 1999). Light is one of the most important environmental factors regulating anthocyanin biosynthesis in plants. Light-regulated anthocyanin synthesis is mostly achieved through the regulation of specific TFs (Allan et al., 2008; Bai et al., 2019; Ni et al., 2019). In many fruit-bearing plants, such as pear, apple, Chinese bayberry, litchi, and eggplant, anthocyanin accumulation involves the light-mediated TF R2R3 MYB (Takos et al., 2006; Niu et al., 2010; Lai et al., 2014; Jiang et al., 2016; Bai et al., 2017). The basic leucine zipper TF HY5, which is regulated by light, directly binds to the gene promoters of R2R3 MYB regulators of anthocyanin biosynthesis to regulate anthocyanin biosynthesis (Shin et al., 2013; Jiang et al., 2016; An et al., 2017; Qiu et al., 2019). In *Arabidopsis*, the expression of anthocyanin biosynthesis-related genes, including production of anthocyanin pigment 1 (*PAP1*), *PAP2*, *TT8*, *GL3*, and *EGL3*, is induced by light (Cominelli et al., 2008). In *Arabidopsis*, the WD40-repeat protein TTG1 forms a complex with bHLH (GL3, EGL3, or TT8) and R2R3 MYB (PAP1, PAP2, MYB113, and MYB114) TFs to regulate anthocyanin synthesis (Zhang et al., 2003; Gonzalez et al., 2008). Low temperature is another important environmental factor that affects anthocyanin biosynthesis. MdbHLH3 binds to the MYC-binding site in the *MdMYB1* promoter to induce anthocyanin accumulation in apple under low temperature (Xie et al., 2012).

The effects of environmental factors on the molecular mechanism of anthocyanin synthesis in *D. candidum* have not been fully elucidated. This study aimed to clarify the molecular mechanism of light-induced anthocyanin biosynthesis regulation in *D. candidum*. To this end, we grew plants under two different light intensities. The WD40-repeat TF DcTTG1 was found to respond to light signals and to regulate anthocyanin biosynthesis. These results provide new insights in the molecular basis of anthocyanin biosynthesis in *D. candidum*.

## Materials and Methods

### Plant Materials and Growth Conditions

*Dendrobium candidum* (cultivar JXH-1) tissues were cultured at the Institute of Medicinal Plants, Sanming Academy of Agricultural Sciences (Sanming, Fujian, China). Plant tissues were cultured in Murashige & Skoog (MS) medium supplemented with 6-BA (2.0 mg/L), sucrose (20 g/L), and agar (6 g/L), pH 5.8, under a 16-h light/8-h dark cycle, at 24°C. For light treatments, 3-cm-high seedlings were transferred to ½ MS medium and cultured under a 16-h light/8-h dark cycle at 24°C. The light intensity of fluorescent lamps (NH-GP-1200R, Nonghui, Shanghai, China) was set to 1,000 lx or 4,000 lx. Seedlings were collected after 30 days of light exposure. Tobacco (*Nicotiana benthamiana*) and wild-type (WT) and *ttg1-13* mutant *A. thaliana* (ecotype Columbia) plants were grown in a mixed matrix of vermiculite/perlite/peat soil (1:1:1, v/v/v) or on MS medium in a greenhouse under a 16-h light/8-h dark cycle and a relative humidity of 40–60%, at 22°C.

### Quantification of Anthocyanins

The total anthocyanin content of *Arabidopsis* leaves was extracted and determined using previously described methods (Zhao and Dixon, 2009; Zhao et al., 2011), using cyanidin 3-*O*-glucoside as a standard. Three biological replicates were analyzed.

*Dendrobium candidum* samples were ground into a fine powder in liquid nitrogen immediately after collection. One hundred milligram of the powder was extracted with 80% methanol aqueous solution containing 0.5% formic acid in a centrifuge tube under shaking for 2 min followed by ultrasonication at room temperature for 30 min. After the extraction, the mixture was centrifuged at 12000 rpm for 10 min. The supernatant liquid was transferred into a new tube and subjected to UPLC-MS/MS, using a 30A UPLC system with a phenylethyl chromatographic column, column temperature of 40°C, flow rate of 0.25 ml/min, and a total injection volume of 30 μl. Three biological replicates were analyzed for each sample.

### Transcriptome Sequencing

RNA was isolated from plant tissue samples using an RNA isolation kit (Aidlab, Beijing, China). RNA quantity and integrity were assessed using a NanoDrop ND-1000 spectrophotometer (NanoDrop Technologies, Wilmington, DE, United States) and an Agilent 2100 Bioanalyzer (Agilent Technologies, Santa Clara, CA, United States), respectively. cDNA libraries were constructed with the NEB Next® Ultra™ RNA Library Prep Kit (New England Biolabs, Ipswich, MA, United States). The cDNA libraries were sequenced on the HiSeq4000 platform (Illumina, San Diego, CA, United States) at Biomarker Technology (Beijing, China). Three biological replicates were analyzed for each sample type. Gene expression levels were analyzed according to the “fragments per kilobase of transcript per million fragments mapped” method. The DESeq R package was used to identify differentially expressed genes (DEGs) in RDc4000 vs. RDc1000. Gene Ontology (GO) enrichment of the DEGs was analyzed using the GOseq R package (Young et al., 2010). Kyoto Encyclopedia of Genes and Genomes (KEGG) pathway enrichment was investigated using the KOBAS tool (Xie et al., 2012). The sequencing data have been submitted to NCBI Sequence Read Archive database under accession numbers SRR13577108, SRR13577107, SRR13577106, SRR13577105, SRR13577104, and SRR13577103.^1^

### Phylogenetic Analysis and Subcellular Localization

Sequences of TTG1-like proteins were downloaded from the National Center for Biotechnology Information database. MEGA X was used for phylogenetic analysis of the TTG1-like proteins. *Dendrobium candidum* DNA was extracted using a DNA isolation kit (Aidlab). Using primers listed in Supplementary Table S1, a *TTG1* sequence was cloned and a fusion vector DcTTG1::YFP was constructed. DcTTG1::YFP was transformed into *Agrobacterium* GV3101 for injection into tobacco (*N. benthamiana*) leaf epidermal cells. 4,6-Diamidino-2-phenylindole (DAPI) was used to stain the nuclei. Fluorescence signals were acquired using a confocal laser scanning microscope (model SP8; Leica Microsystems).

### RNA Extraction, cDNA Synthesis, and Quantitative Reverse-Transcription PCR

RNA was extracted from *D. candidum* tissue samples using an RNA isolation kit (Aidlab). The RevertAid Premium First Strand cDNA Synthesis Kit (Fermentas/Thermo Fisher Scientific, Rochester, NY, United States) was used for cDNA synthesis. Quantitative reverse transcription PCRs (RT-qPCRs) were run on a 7500 Fast Quantitative PCR system (Applied Biosystems) using TB Green® *Premix Ex Taq*™ (Takara). *DcACT* was selected as a reference gene to normalize target gene expression according to the 2^−ΔΔCT^ method. Three biological replicates were analyzed. The primers used for RT-qPCR are listed in Supplementary Table S2.

### Y1H Assay

The coding region of *DcTTG1* was ligated into the pGADT7 vector and promoter fragments of several target genes related to anthocyanin biosynthesis were ligated into the pHIS2 vector (Clontech, Palo Alto, CA, United States). Fusion products of the pGADT7 and pHIS2 vectors were transformed into yeast strain Y187 cells (Clontech, Palo Alto, CA, United States). The cells were cultured in synthetic defined (SD) medium –Leu/–Trp/–His with different concentrations of 3-amino-1,2,4-triazole (3-AT, mM) to prevent background histidine leakage of the pHIS2 vector at 28°C for 3 days. Empty pGADT7 vector was used as a control. The primers used for vector construction are listed in Supplementary Table S3.

### Transcriptional Activity Assay

Transcriptional activity was analyzed according to previous reports (Liu et al., 2016; Jia et al., 2018). The coding sequence of *DcTTG1* was fused into the 35S-LUC-GUS vector driven by the 35S promoter. Promoter fragments of *DcCHs1*, *DcCHS2*, *DcCHI*, *DcF3H*, *DcF3'H*, *DcDFR1*, *DcDFR2*, *DcANS1*, and *DcUFGT* were cloned into the 35S-LUC-GUS vector driven by the GUS reporter. The internal standard was LUC. The fusion vectors were transformed into *Agrobacterium* GV3101 cells. Single clones were cultured in liquid medium (10 mM MgCl_2_, 10 mM MES, and 100 μM acetosyringone) under shaking until an optical density at 600 nm of 0.5 was reached. Then, the *Agrobacterium* cells were injected into tobacco leaves. GUS and LUC activities were measured after 4 days. At least five biological replicates were included in each assay. The primers used for vector construction are listed in Supplementary Table S4.

## Results

### Red Pigmentation in RDc4000 Is Caused by Anthocyanin Accumulation

**Figure 1:**
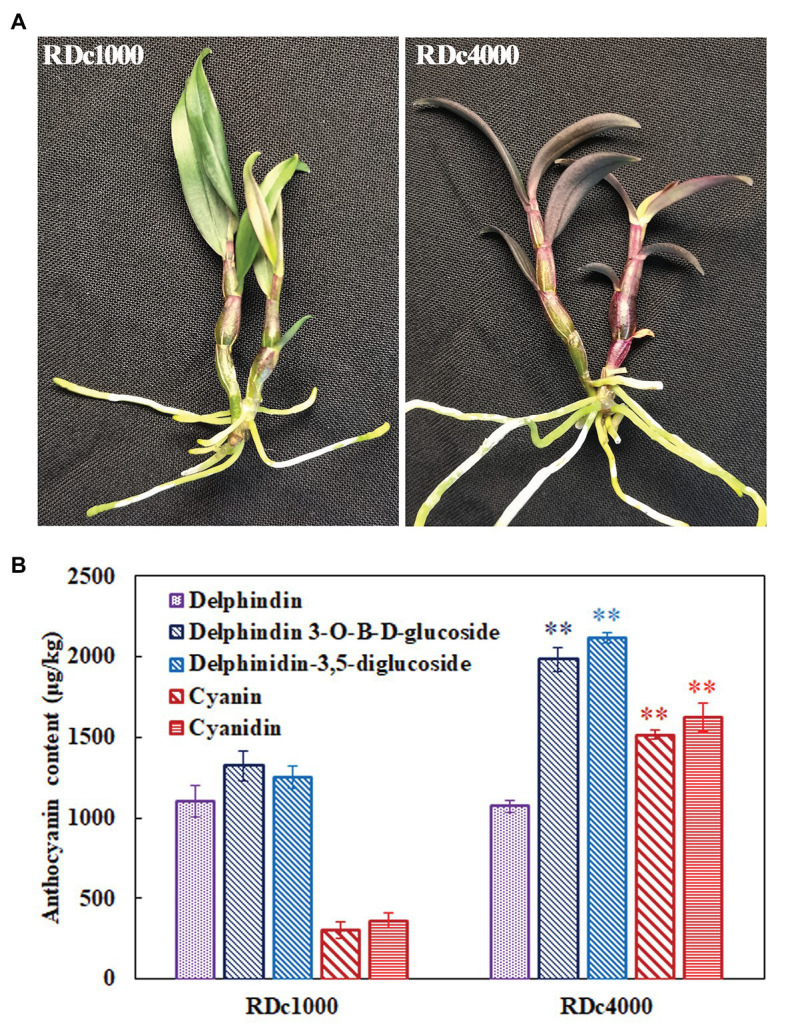
Photographs and anthocyanin contents of *Dendrobium candidum* under two different light treatments. **(A)** Photographs of RDc1000 (red *D. candidum* seedlings under 1,000 lx) and RDc4000 (red *D. candidum* seedlings under 4,000 lx). **(B)** Anthocyanin composition in RDc1000 and RDc4000, including delphindin, delphindin-3-*O*-B-d-glucoside, delphindin-3,5-diglucoside, cyanin, and cyanidin, as assessed by UPLC-MS/MS. All data represent the mean ± SE of three biological replicates. ^∗∗^*p* < 0.01, Student’s *t*-test.

The phenotypes of *D. candidum* seedlings grown under 1,000 lx (RDc1000) and 4,000 lx (RDc4000) are shown in Figure 1A. RDc1000 stems had red pigmentation. The stems of RDc4000 were darker than those of RDc1000, and the leaves were dark red. In general, RDc4000 had more red pigment than RDc1000, indicating that higher-intensity light stimulates the accumulation of red pigment. Analysis of the anthocyanin contents to reveal the type and quantity of red pigmentation under the two light conditions revealed that *D. candidum* contained delphindin, delphindin-3-*O*-B-d-glucoside, delphindin-3,5-diglucoside, cyanin, and cyanidin under both conditions, but the contents of these five compounds were different. Delphindin-3-*O*-B-d-glucoside was the most abundant in RDc1000 and delphindin-3,5-diglucoside was the most abundant in RDc4000; however, the content of delphindin did not differ between the two light conditions. Notably, the contents of delphindin-3-*O*-B-d-glucoside, delphindin-3,5-diglucoside, cyanin, and cyanidin were significantly higher in RDc4000 than in RDc1000. These results indicated that a higher light intensity promotes the production of red pigment, mainly of the anthocyanin compounds delphindin-3-*O*-B-d-glucoside, delphindin-3,5-diglucoside, cyanin, and cyanidin, in *D. candidum*.

### Transcriptome Analysis of RDc1000 and RDc4000

**Figure 2:**
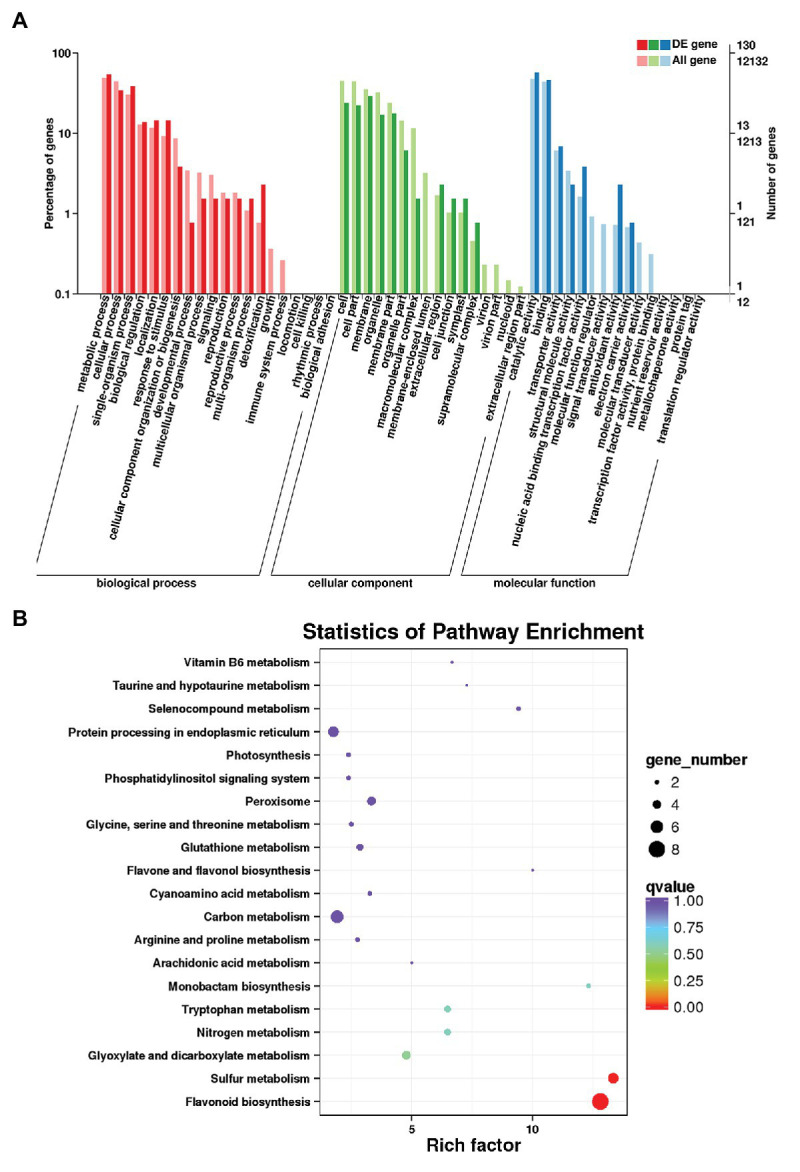
Transcriptome analysis of *D. candidum* stems. **(A)** Gene Ontology (GO) enrichment analysis results for differentially expressed genes (DEGs) between RDc1000 and RDc4000. **(B)** Kyoto Encyclopedia of Genes and Genomes (KEGG) enrichment analysis of the DEGs.

Transcript-level differences in *D. candidum* under the different light intensities were analyzed by transcriptome sequencing of RDc1000 and RDc4000. The original sequencing data were transformed into raw reads *via* base calling. The total number of bases in the six libraries was 43.62 G, and the total number of bases in each sample was greater than 6.5 Gb. The Q20 and Q30 scores for each sample were above 97 and 94%, and the GC content was relatively consistent, around 46% (Supplementary Table S5). The mapping rate, i.e., the percentage of mapped reads in clean reads, the most directly reflects sequencing data utility. The mapping rate of the samples ranged from 90.5 to 91.08%. The percentage of uniquely and multi-mapped reads in clean reads ranged from 87.75 to 88.36% and from 2.38 to 2.74%, respectively (Supplementary Table S6). These findings indicated that the data met the quality requirements for analysis. In total, 130 unigenes could be assigned to 51 Gene Ontology (GO) terms in the cellular component, molecular function, and biological process categories. In the cellular component category, DEGs in RDc4000 vs. RDc1000 were mainly related to the plasma membrane, cell, cell parts, and organelles. In the molecular function category, most DEGs were related to catalytic activity and binding. In the biological process category, DEGs were mainly involved in metabolic processes, single-organism processes, and cellular processes (Figure 2A).

In total, 258 DEGs were found, 152 of which were upregulated and 106 of which were downregulated in RDc4000 vs. RDc1000. Among these, there were 22 TF genes, comprising eight upregulated and 14 downregulated genes, including *MYB* (two genes), *bHLH* (two genes), *WRKY* (two genes), *AP2/ERF* (two genes), *bZIP* (two genes), *NAC* (two genes), and the WD40-repeat TF gene, *DcTTG1* (Supplementary Table S7). Among the 22 differentially expressed TF genes, *DcTTG1* was upregulated the most strongly in RDc4000 vs. RDc1000 (Supplementary Table S7). Therefore, we speculated that DcTTG1 may be involved in the synthesis of anthocyanins. To identify the metabolic pathways involved in light-induced anthocyanin biosynthesis, 60 DEGs were subjected to KEGG pathway enrichment analysis, and the 20 most enriched pathways are shown in Figure 2B. In these pathways, we found eight DEGs involved in flavonoid biosynthesis, including genes encoding dihydroflavonol-4-reductase, naringenin, 2-oxoglutarate 3-dioxygenase, flavanone 3-dioxygenase, and leucoanthocyanidin dioxygenase, which are key genes in anthocyanin biosynthesis (Supplementary Table S8). These results indicated that these eight genes are the most important genes in the pathway of light-induced anthocyanin biosynthesis.

### Expression Analysis and Characterization of DcTTG1

**Figure 3:**
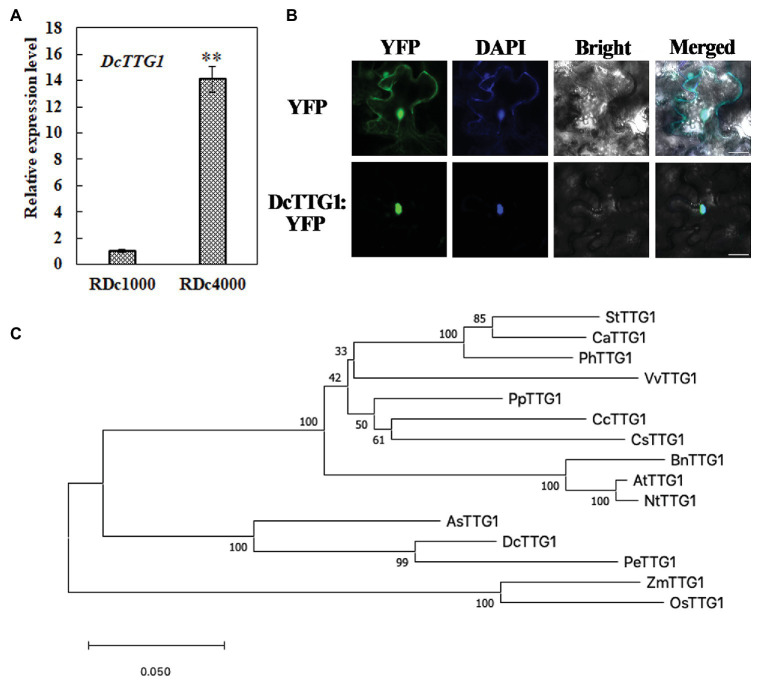
Phylogenetic, expression, and subcellular localization analyses of DcTTG1. **(A)** Relative expression of *DcTTG1* in RDc1000 and RDc4000 as determined by quantitative reverse transcription PCR (RT-qPCR). DcACT was used as reference gene for normalization. Data represent the mean ± SE of three biological replicates. ^∗∗^*p* < 0.01, Student’s *t*-test. **(B)** Subcellular localization of DcTTG1. 4,6-Diamidino-2-phenylindole (DAPI) was used as a nuclear marker. The merged image shows the colocalization of the YFP fluorescence signal and the DAPI fluorescence signal. Bar = 20 μm. **(C)** Phylogenetic analysis of TTG1-like proteins. The following proteins were used to construct the phylogenetic tree: StTTG1 (NP_001305551.1), CaTTG1 (XP_016564215.1), PhTTG1 (AAC18914.1), VvTTG1 (CAN67365.1), PpTTG1 (ACQ65867.1), CcTTG1 (AMQ26245.1), CsTTG1 (NP_001306987.1), BnTTG1 (NP_001303154.1), AtTTG1 (CAC10524.1), NtTTG1 (ACJ06978.1), AsTTG1 (PKA51013.1), PeTTG1 (XP_020583423.1), ZmTTG1 (NP_001310302.1), and OsTTG1 (KAB8088430.1).

To better understand the role of this TF in anthocyanin production in *D. candidum* under the different light regimens, it was studied in depth. The *DcTTG1* sequence was cloned from *D. candidum* cDNA. The relative expression of *DcTTG1* in RDc1000 and RDc4000 was analyzed by RT-qPCR. The results are shown in Figure 3A. *DcTTG1* expression in RDc4000 was 14 times higher than that in RDc1000, indicating that it is induced with increasing light intensity (Figure 3A). To study its regulation in anthocyanin biosynthesis, we conducted subcellular localization analysis using a Pro35S::DcTTG1-YFP vector construct that was transfected into *Agrobacterium* cells, which were injected into tobacco leaf epidermal cells. The fluorescence signal of Pro35S::DcTTG1-YFP was observed in the nucleus and overlapped with that of the nuclear dye, DAPI, indicating that DcTTG1 is located in the nucleus in *D. candidum* (Figure 3B). Next, we conducted phylogenetic analysis of DcTTG1 and TTG1-like proteins of other species (Figure 3C). DcTTG1 and PeTTG1 from *Phalaenopsis equestris* were in the same branch and were closely related. Together with the transcriptional expression differences after the different light treatments, this suggested that DcTTG1 plays an important role in light-regulated anthocyanin biosynthesis.

### Expression Analysis of Key Anthocyanin Biosynthetic Genes

Anthocyanin accumulation affects plant pigmentation, resulting in different plant colors. To determine the effect of the different light intensity regimens on anthocyanin metabolism, the expression of 10 key anthocyanin biosynthetic genes, including *DcCHS1*, *DcCHS2*, *DcCHI*, *DcF3H*, *DcF3'H*, *DcF3'5'H*, *DcDFR1*, *DcDFR2*, *DcANS1*, and *DcUFGT*, was analyzed by RT-qPCR in RDc1000 and RDc4000 plants. The mRNA levels of *DcCHS1*, *DcCHS2*, *DcF3H*, *DcF3'H*, *DcDFR1*, *DcDFR2*, and *DcANS1* were significantly higher in RDc4000 than in RDc1000. *DcDFR1* was the most strongly induced, by approximately 27 times, followed by *DcF3H*. These results indicated that, the stronger the light intensity, the higher the expression of DFR, which greatly promotes anthocyanin accumulation in plants. These seven genes included early as well as late anthocyanin biosynthetic genes, indicating that high-intensity light has a significant effect on both the early and late stages of anthocyanin synthesis.

### DcTTG1 May Regulate the Expression of *DcCHS2*, *DcCHI1*, *DcF3H*, and *DcF3'H* by Binding to Their Promoters

**Figure 4:**
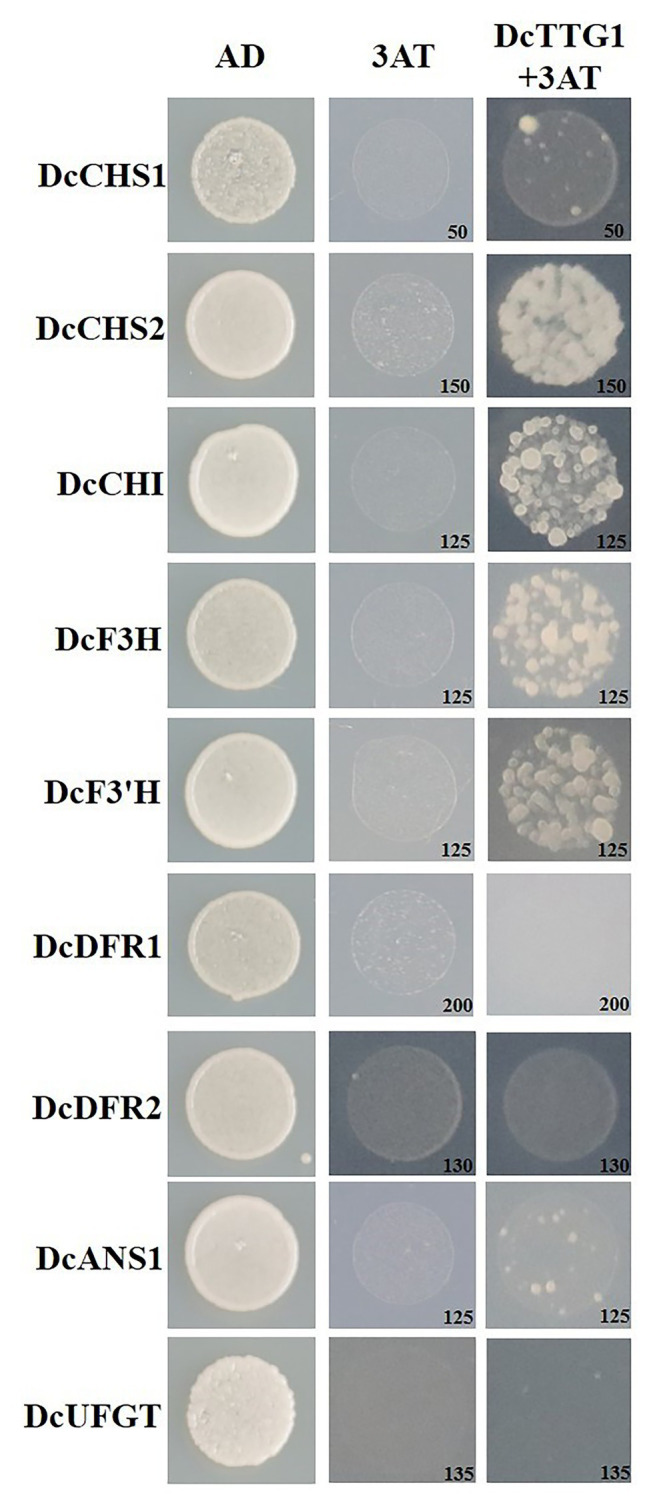
Y1H assays of DcTTG1 and anthocyanin biosynthesis-related gene promoters. The *DcTTG1* sequence and promoter fragments of nine anthocyanin biosynthetic genes were cloned into pGADT7 and pHIS2 vectors, respectively. The plasmids were transformed into Y187 yeast cells (Clontech Laboratories, Mountain View, CA, United States) that were grown on SD (–Leu/–Trp/–His) medium at 28°C for 3 days. The optimal 3-AT concentration (in mM) to suppress background histidine leakiness after addition to the (–Leu/–Trp/–His) medium is indicated in the lower-right corner of each picture.

TTG1 is a WD40-repeat TF that can bind to the promoter regions of target genes to regulate their transcription. A Y1H assay was used to analyze the regulation of target genes by DcTTG1. The promoter sequences of nine anthocyanin biosynthetic genes, *DcCHs1*, *DcCHS2*, *DcCHI*, *DcF3H*, *DcF3'H*, *DcDFR1*, *DcDFR2*, *DcANS1*, and *DcUFGT*, were cloned separately to verify whether DcTTG1 has a regulatory effect on these genes. An appropriate concentration of 3-AT in the yeast growth medium (SD/–Leu/–Trp/–His) inhibited the self-activation of all target genes. The assay results suggested that DcTTG1 can bind to the promoters of *DcCHS2*, *DcCHI*, *DcF3H*, and *DcF3'H* (Figure 4).

**Figure 5:**
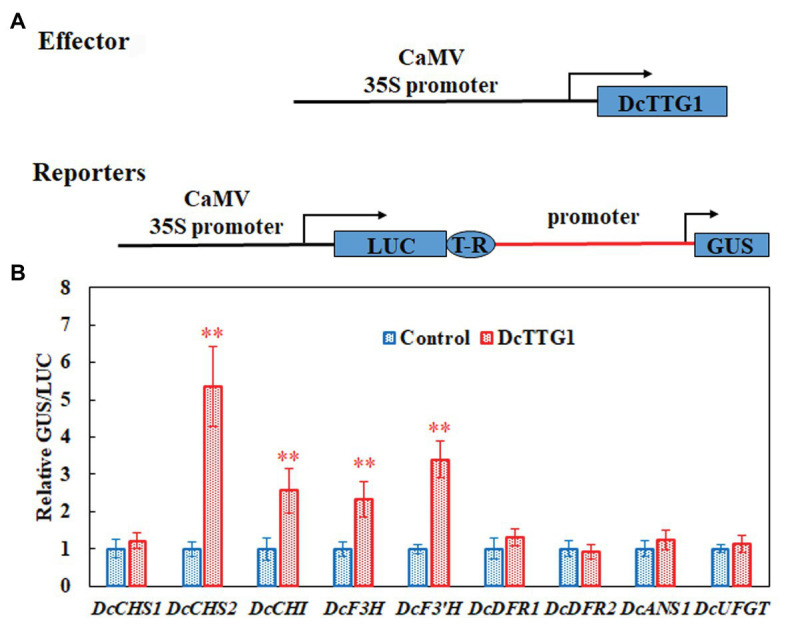
DcTTG1 can directly bind to the promoters of anthocyanin biosynthetic genes. **(A)** Effector-reporter systems were used to determine the ability of DcTT1 to activate the promoters of anthocyanin biosynthetic genes in tobacco leaves. The reporter systems contained the *GUS* reporter gene driven by the promoters of anthocyanin biosynthetic genes, as well as *LUC* driven by CaMV35S for standardization. The effector systems contained *DcTTG1* driven by CaMV35S. Boxes, various DNA sequences. T-R, terminator. **(B)** Transcriptional activity of DcTTG1 on the promoters of anthocyanin biosynthetic genes as indicated by the reporter assay. Empty vector was used as a control, and the GUS/LUC ratio in the transformed leaves was set to 1. Data represent the mean ± SE of five biological replicates. ^∗∗^*p* < 0.01, Student’s *t*-test.

To verify the Y1H assay results, we used LUC/GUS analysis to detect the effect of DcTTG1 on the activation of key anthocyanin biosynthetic gene promoters. We cloned anthocyanin biosynthetic gene promoters, which we used to drive the GUS reporter gene, and DcTTG1 was driven by the CaMV35S promoter (Figure 5A). Transient expression of the reporter system in tobacco revealed that DcTTG1 activated reporter gene expression driven by the promoters of *DcCHS2*, *DcCHI*, *DcF3H*, and *DcF3'H* (Figure 5B), corroborating that DcTTG1 regulates the expression of these four genes. Thus, DcTTG1 modulates anthocyanin synthesis by regulating the expression of anthocyanin synthesis-related target genes under different light intensities.

### Phenotype Rescue of the *Arabidopsis ttg1-13* Mutant

**Figure 6:**
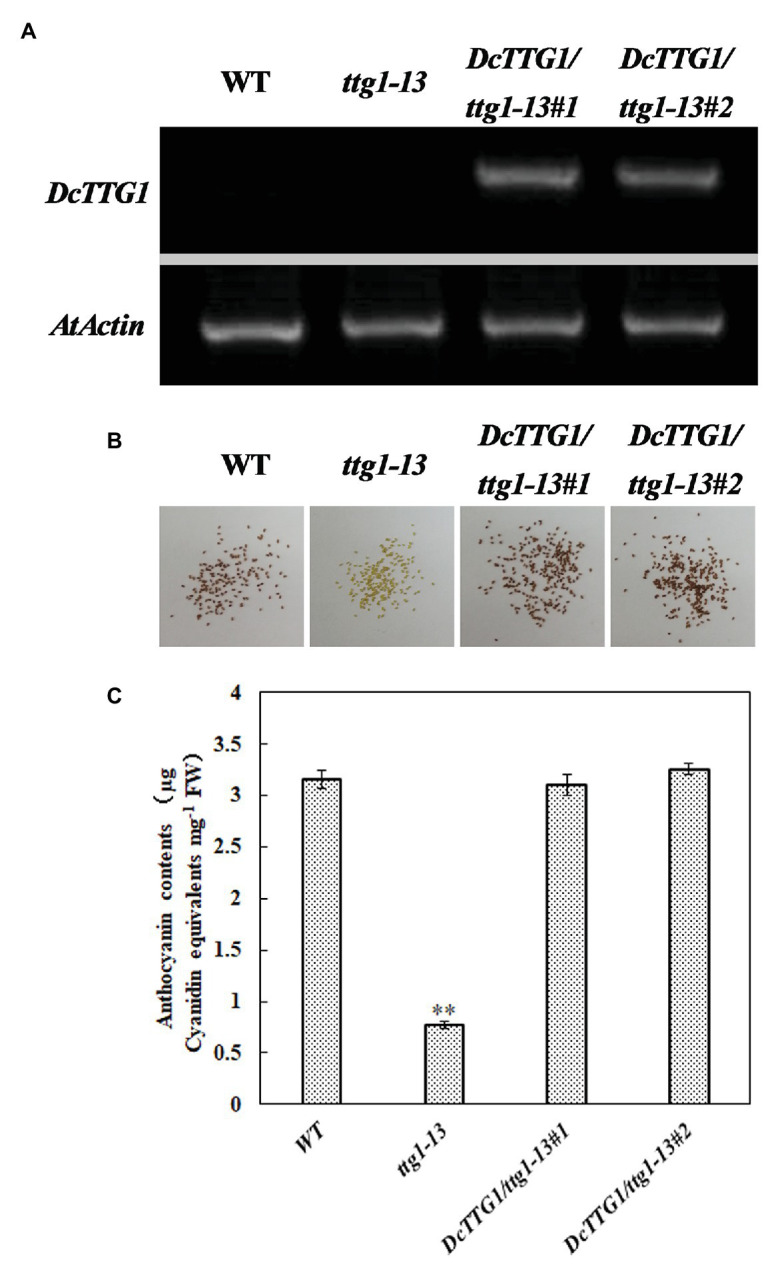
Genetic complementation of the *Arabidopsis ttg1-13* mutant by DcTTG1. **(A)** *DcTTG1* transcripts in *Arabidopsis* young seeds. **(B)** Restored seed pigments in *ttg1-13* after overexpression of *DcTTG1*. **(C)** Quantification of anthocyanins in the leaves of WT, *ttg1*, *DcTTG1/ttg1-13#1*, and *DcTTG1/ttg1-13#2*. Data represent the mean ± SE of three biological replicates. ^∗∗^*p* < 0.01, Student’s *t*-test.

Establishing transgenic *D. candidum* lines is generally difficult and time-consuming. Therefore, we used the model plant *Arabidopsis* to verify the function of DcTTG1 in the regulation of anthocyanin biosynthesis. To further study the possible function of *DcTTG1* as a regulator of anthocyanin biosynthesis, we overexpressed *DcTTG1* driven by the CaMV35S promoter in the *Arabidopsis ttg1-13* mutant, which is defect in seed coat anthocyanin pigmentation. We obtained 18 *DcTTG1* overexpression lines and analyzed lines *DcTTG1/ttg1-13#1* and *DcTTG1/ttg1-13#2*. The *DcTTG1* expression levels in these lines were significantly higher than those in WT plants (Figure 6A), indicating successful overexpression. *DcTTG1* overexpression restored the *ttg1-13* mutant phenotype (Figure 6B; Supplementary Figure S1) as well as leaf anthocyanin pigmentation (Figure 6C), suggesting that DcTTG1 has a similar function as *Arabidopsis* TTG1.

## Discussion

This study revealed that the contents of delphindin-3-*O*-B-d-glucoside, delphindin-3,5-diglucoside, cyanin, and cyanidin were significantly increased in RDc4000 compared with RDc1000. Transcriptome sequencing and RT-qPCR showed that the WD40-repeat TF DcTTG1 was significantly more strongly expressed in RDc4000 than in RDc1000. Phylogenetic analysis and genetic complementation revealed the functional conservation of DcTTG1 and its orthologs in other plant species. Y1H and transcriptional activation assays indicated that DcTTG1 regulates *DcCHS2*, *DcCHI*, *DcF3H*, and *DcF3'H* expression by binding to their promoters. Thus, DcTTG1 may regulate anthocyanin biosynthesis in *D. candidum* under different light intensities.

**Figure 7:**
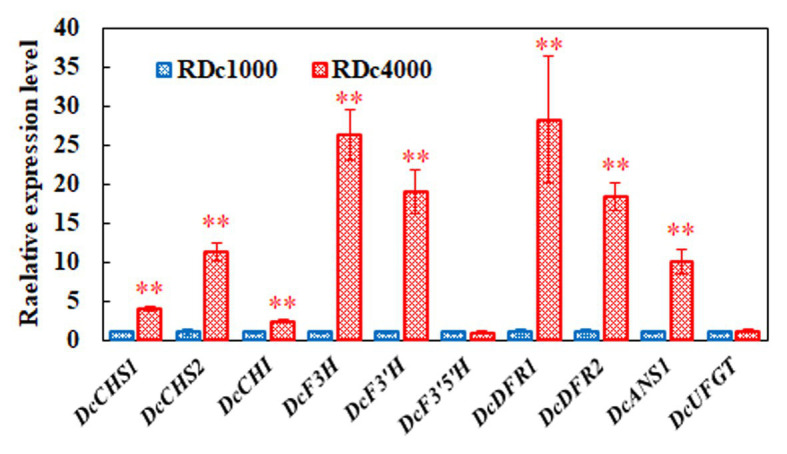
Expression of key anthocyanin biosynthetic genes. *DcACTIN* was used as reference gene for normalization. All gene expression levels in RDc1000 were set to 1. Error bars represent the mean ± SE of three biological replicates. ^∗∗^*p* < 0.01, Student’s *t*-test.

Anthocyanin compositional and content analyses showed that the content of delphindin did not significantly differ between RDc1000 and RDc4000 (Figure 1B). In the anthocyanin biosynthetic pathway, the formation of delphindin requires F3*'*5*'*H catalysis (Matus et al., 2009). *DcF3'5'H* expression did not significantly differ between RDc1000 and RDc4000, which may explain the lack of a significant difference in delphindin content. However, the contents of delphindin-3-*O*-B-d-glucoside and delphindin-3,5-diglucoside were significantly different between RDc1000 and RDc4000, which may be due to significant increases in *DcDFR1*, *DcDFR2*, and *DcANS1* expression in RDc4000 (Figure 7). The cyanin and cyanidin contents were significantly higher in RDc4000 than in RDc1000 (Figure 7). The formation of cyanin and cyanidin requires F3'H catalysis (Rinaldo et al., 2015), and DcF3*'*H expression was significantly higher in RDc4000 than in RDc1000 (Figure 7). Light provides energy for plant growth and development (Lau and Deng, 2010). It regulates plant secondary metabolism, including anthocyanin and flavonoid synthesis (Zhang et al., 2018; An et al., 2020). Different light intensities have different effects on anthocyanin accumulation, which is positively correlated with light intensity in many plant species (Jaakola, 2013; Zhang et al., 2018).

In *Arabidopsis*, *AtTTG1* is expressed in the dark, but its expression increases immediately after illumination and then remains relatively stable over time (Cominelli et al., 2008). In our study, *DcTTG1* expression increased significantly with increasing light intensity (Figure 3A). In Brassica, anthocyanins accumulate more strongly under LED irradiance with a photosynthetic photon flux of 220 μmol m^–2^ s^−1^ than under a flux of 330 or 440 μmol m^–2^ s^−1^ (Samuoliene et al., 2013). Within a certain range, anthocyanin accumulation gradually increases with increasing light intensity, but after a certain intensity, the amount of anthocyanin does not further increase due to photoprotection (Logan et al., 2015). Anthocyanin levels in lychee peels decreased rapidly after shading treatment, but anthocyanin biosynthesis resumed soon after the fruits were exposed to light again (Zhang et al., 2016). This shows that light can induce anthocyanin formation and is an important environmental factor affecting anthocyanin biosynthesis. Similar findings have been reported for Chinese bayberry (Niu et al., 2010), apple (Li et al., 2012), and grape berry (Azuma et al., 2012).

Transcriptome sequencing analysis was performed on RDc1000 and RDc4000 to study the relationship between light intensity and anthocyanin accumulation. Among the TFs identified by transcriptome sequencing, MYB, bHLH, and WD40 all showed significant differences in expression, indicating that MBW may be involved in anthocyanin biosynthesis induced by high light intensity in *D. candidum*. Transcriptome analysis of light-regulated anthocyanin biosynthesis in the pericarp of Litchi identified 76 TFs that regulate anthocyanin synthesis in response to light. Among these TFs, SQUAMOSA promoter binding protein-like constituted the largest population (21.1%), followed by MYB (19.7%), homologous domain leucine zipper protein (ATHB, 17.1%), and WD40 (13.2%; Zhang et al., 2016). Thus, light intensity is an important environmental factor in regulating anthocyanin biosynthesis.

The expression levels of *DcCHI*, *DcF3'5'H*, and *DcUFGT* did not differ significantly between the two light treatments, indicating that these genes are less responsive to different light intensities in *D. candidum*. Interestingly, our and previous findings indicate that the feedback regulation of anthocyanin synthetic genes in response to light varies among plant species. Leaves of *Lactuca sativa* L. var. capitata were green under 40 mmol m^−2^ s^−1^, but red under 100 mmol m^−2^ s^−1^, and the expression of *LsCHI* was upregulated (Zhang et al., 2018). However, in a study of light-induced regulation of anthocyanin biosynthesis in litchi fruit, the activation and expression of *LcUFGT* promoted the accumulation of anthocyanins and enhanced fruit redness (Zhang et al., 2016). Anthocyanin biosynthetic genes of different species differentially respond to light, which emphasizes the importance of studying the effects of different light treatments on anthocyanin synthesis in *D. candidum*.

DcTTG1 modulates anthocyanin synthesis by regulating the expression of anthocyanin synthesis-related target genes under different light intensities. In this study, no direct binding of TTG1 to late anthocyanin biosynthetic gene promoters was observed, which indicates that DcTTG1 has multiple modes of regulating key genes in anthocyanin biosynthesis. Gene regulatory networks in higher plants require the coordinated action of many gene interactions. Among them, the most widely studied is the regulation of anthocyanin biosynthesis by the MBW complex. TTG1 forms complexes with the R2R3 MYB TFs PAP1, PAP2, MYB113, MYB114, or TT2 and the bHLH TFs GL3, EGL3, or TT8, to form MBW (Xu et al., 2015). Research in *Arabidopsis* has shown that the TTG1-dependent MBW complex can directly bind to the promoters of *TTG2*, *TT8*, *F3'H*, *DFR*, *ANS*, *UGT79B1*, *UGT75C1*, *5MAT*, and *BLT* (Wei et al., 2019). Therefore, it can be inferred that DcTTG1 alone cannot regulate the late anthocyanin biosynthetic genes, whereas different TTG1-dependent MBW complexes can simultaneously regulate the early and late anthocyanin biosynthetic genes. The different MBW complexes formed by TTG1 have diverse regulatory roles in anthocyanin biosynthesis. The TTG1-TT8/GL3-PAP1/PAP2/MYB113/MYB114 complex can activate the expression of late biosynthetic genes, including dihydroflavonol 4-reductase (*DFR*) and phthalate synthase (*ANS*), to affect anthocyanin biosynthesis (Shi and Xie, 2014). There is no relevant research on the characteristic roles of DcTTG1 and the DcTTG1-dependent MBW complex in the regulation of anthocyanin biosynthetic structural gene expression, especially under different light conditions. Our findings may contribute to improving plant production through the regulation of certain desired metabolic compounds.

In conclusion, we identified and isolated a WD40-repeat TF, DcTTG1, as a novel regulator of light-regulated anthocyanin accumulation in *D. candidum*. DcTTG1 regulates anthocyanin biosynthesis in various ways. The TF binds to the promoters of *DcCHS2*, *DcCHI*, *DcF3H*, and *DcF3'H*, which are anthocyanin biosynthesis-related genes. DcTTG1 restored the *Arabidopsis ttg1-13* mutant phenotype, which is defect in seed coat anthocyanin pigmentation, suggesting that it has a similar function as *Arabidopsis* TTG1. We believe that the findings of this study will help improve the understanding of the mechanism of light-induced anthocyanin synthesis and accumulation.

## Data Availability Statement

The transcriptome data of this study have been uploaded to NCBI Sequence Read Archive database (https://ncbiinsights.ncbi.nlm.nih.gov/tag/sra/), the accession number is PRJNA694810.

## Author Contributions

NJ, FW, and BF designed the study. NJ, JW, YW, JL, JJ, JS, PY, and PW performed the experiments. NJ, JW, FW, and BF wrote the manuscript. All authors contributed to the article and approved the submitted version.

### Conflict of Interest

The authors declare that the research was conducted in the absence of any commercial or financial relationships that could be construed as a potential conflict of interest.

## Footnotes

^1^https://ncbiinsights.ncbi.nlm.nih.gov/tag/sra/
