# Supplementary material for: The Light-Induced WD40-Repeat Transcription Factor DcTTG1 Regulates Anthocyanin Biosynthesis in Dendrobium candidum
Source: Front Plant Sci. 2021 Mar 17;12:633333. doi: 10.3389/fpls.2021.633333 (PMC8010245; doi:10.3389/fpls.2021.633333)
Supplement: Supplementary file 1 [file Data_Sheet_1.docx]

Supplementary Material

**Table S1** Primers used for subcellular localization analysis and genetic complementation.

| **Primer name** | **Primer sequence** |
| --- | --- |
| DcTTG1-1-F | 5'-ATG CTA CAA TTA CCA ACC ACG CAC G-3' |
| DcTTG1-2-R | 5'-CCT ACG CGT AAC CCT GAG CAA CTG CAC CTT GTT TGA G-3' |
| DcTTG1-3-R | 5'-CCT ACG CGT TCA AAC CCT GAG CAA CTG CAC CTT GTT TGA G-3' |

**Table S2** Primers used for RT-qPCR analysis.

| **Primer name** | **Primer sequence** |
| --- | --- |
| DcTTG1-4-F | 5'-GGA CAG TAA TCA GGT GGT CGT CCT TG-3' |
| Dc TTG1-5-R | 5'-AAC CCT GAG CAA CTG CAC CTT GTT TGA G-3' |
| DcCHS1-5-F | 5'-GAT ACT CGA CCA AGT TGA AAT TAA GCT TGG-3' |
| DcCHS1-6-R | 5'-GAA CGC TGC GTA GCA CAA CAG TTT CTA C-3' |
| DcCHS2-5-F | 5'-CAT TCT AGA TCA GCT GGA CGA GAG AGT G-3' |
| DcCHS2-6-R | 5'-GAA CAC TGC GGA GAA CGA CGG TAT C-3' |
| DcCHI-5-F | 5'-CCC ACA GCT GTT GAT GAA TCT GCA TTA TC-3' |
| DcCHI-6-R | 5'-GAT ACC TTC AAC GTA GGG GAA ACT GG-3' |
| DcF3H-5-F | 5'-GTC TTT CGA TCG CGA CGT TCC AGA AC-3' |
| DcF3H-6-R | 5'-CAT TTA AGA CCT TCG GCT TCG TTG CGG-3' |
| DcF3'H-5-F | 5'-GCT TTC GAT CGC GAC GTT TCA GAA C-3' |
| DcF3'H-6-R | 5'-CTA AGC ATT TTG TCC TGC TGC TCC-3' |
| DcF3'5'H-5-F | 5'-GAA ATG ATT TCG AGC TCA TAC CGT TCG-3' |
| DcF3'5'H-6-R | 5'-CTT CCG GCA AAC TCC AAT CAA AG-3' |
| DcDFR1-5-F | 5'-CAA AGA CCC TGA GAA TGA AGT GAT ACA AC-3' |
| DcDFR1-6-R | 5'-CGT ACG TAC CCA ACC GGT CAT CTT GAC-3' |
| DcDFR2-5-F | 5'-GTG CGT AAG CTT CTC TTC AAA GAA GTT G-3' |
| DcDFR2-6-R | 5'-TCA CTT AAC AGC AAT CTG TTC TTT ATT CTC CTC-3' |
| DcANS-5-F | 5'-CGG ATT TCT TGG GCT GTT TTC TGC-3' |
| DcANS-6-R | 5'-CTA CAC AAC CGC CTT GTC TCC GCT TC-3' |
| DcUFGT-5-F | 5'-GAG AAG CTT TTG GTG GAG GTA TTG CAA ATT G-3' |
| DcUFGT-6-R | 5'-CAA TTT CTC TAT CTC ATT ATG CGA TGA CCC-3' |
| DcActin-1-F | 5'-TCC CAA GGC AAA CAG AGA AA-3' |
| DcActin-2-R | 5'-GGC CAC TAG CAT ATA GGG AAA G-3' |
| AtActin-1-F | 5'-CTC AAT CAT GAA GTG TGA TGT GG-3' |
| AtActin-2-R | 5'-GAT CAA TTT TTA CCT GCT GGA ATG-3' |

**Table S3** Primers used for the yeast one-hybrid assay.

| **Primer name** | **Primer sequence** |
| --- | --- |
| DcTTG1-6-F | 5'-CCT CAT ATG CTA CAA TTA CCA ACC ACG CAC G-3' |
| DcTTG1-7-R | 5'-CCT GGA TCC TCA AAC CCT GAG CAA CTG CAC CTT GTT TGA G-3' |
| DcCHS1-1-F | 5'-CCT GAA TTC GCC TGA AAT TTA GGC CCA TAA ACT AAA CAA C-3' |
| DcCHS1-2-R | 5'-CCT GAG CTC TAA GTA ACT CCC TCC CTA TTT CCT CGC-3' |
| DcCHS2-1-F | 5'-CCT GAA TTC GTC TCT TAT TAA TAC CAC ACC CTC TCT C-3' |
| DcCHS2-2-R | 5'-CCT GAG CTC GGT GAA AAG TTT TGT GTT TTG CTT TAG AGG-3' |
| DcCHI-1-F | 5'-GAT GAT GAA ATT TGT ATT TTC ACT TGA AGT TTT AGG GG-3' |
| DcCHI-2-R | 5'-CCT ACG CGT CTG TAA TTC GGG AAC ACC TGC CAC TA-3' |
| DcF3H-1-F | 5'-CCT GAA TTC CCT TAT TTA CCA TCC GAT AGA CGG ACC-3' |
| DcF3H-2-R | 5'-CCT GAG CTC CTC TCT CTC TCT CTT TGT TTG ACG CC-3' |
| DcF3'H-1-F | 5'-CCT GAA TTC GGT GCA ATA TTC GTC CTA AGA AAT TCA TG-3' |
| DcF3'H-2-R | 5'-CCT GAG CTC TGT CTC TCG GCC ATA TTG CCG AAC-3' |
| DcF3'5'H-1-F | 5'-CCT GAA TTC CCA ACT TTA TAG AAA GCC TAC AGC TCTATTTATAGC-3' |
| DcF3'5'H-2-R | 5'-CCT GAA TTC AGT GGA GTT TGT TAG CTT CAA GAG CCT TTC-3' |
| DcDFR1-1-F | 5'-CCT GAA TTC CCA ATT TCA GAC GGA CAT CTT AAA TCT C-3' |
| DcDFR1-2-R | 5'-CCT GAG CTC TTC AGT TCA ACT CAT GCA CAG CAC-3' |
| DcDFR2-1-F | 5'-CCT GAA TTC GAG TTT ACC TTC AAC TCA CTC TCT AGT CTT ATC-3' |
| DcDFR2-2-R | 5'-CCT GAG CTC GCC TTC TCA GCA AGT GTT TTT GAT AGG-3' |
| DcANS-1-F | 5'-CCT GAA TTC GGG AGA TCA AAA GAA GCA TGG CAA AG-3' |
| DcANS-2-R | 5'-CCT GAG CTC TGT GTA TTC TTA GTG AGT AGC TTT GGC TG-3' |
| DcUFGT-1-F | 5'-CCT GAA TTC TCA GTT AAT TGT AAA AGT AAG TAG TTT TTT TG-3' |
| DcUFGT-2-R | 5'-CCT GAG CTC GGC CAG AAA GCT TGA ACC AGA TGG AAG GGA TG-3' |

**Table S4** Primers used for the transcriptional activity assay.

| **Primer name** | **Primer sequence** |
| --- | --- |
| DcTTG1-8-F | 5'-CCT GGA TCC ATG CTA CAA TTA CCA ACC ACG CAC G-3' |
| DcTTG1-9-R | 5'-CCT AGA TCT TCA AAC CCT GAG CAA CTG CAC CTT GTT TGA G-3' |
| DcCHS1-3-F | 5'-CCT GAC GTC GCC TGA AAT TTA GGC CCA TAA ACT AAA CAA C-3' |
| DcCHS1-4-R | 5'-CCT CTC GAG TAA GTA ACT CCC TCC CTA TTT CCT CGC-3' |
| DcCHS2-3-F | 5'-CCT GAC GTC GTC TCT TAT TAA TAC CAC ACC CTC TCT C-3' |
| DcCHS2-4-R | 5'-CCT CTC GAG GGT GAA AAG TTT TGT GTT TTG CTT TAG AGG-3' |
| DcCHI-3-F | 5'-CCT GAC GTC ATT TGT ATT TTC ACT TGA AGT TTT AGG GG -3' |
| DcCHI-4-R | 5'-CCT CTC GAG CTG TAA TTC GGG AAC ACC TGC CAC TA-3' |
| DcF3H-3-F | 5'-CCT GAC GTC CCT TAT TTA CCA TCC GAT AGA CGG ACC-3' |
| DcF3H-4-R | 5'-CCT CTC GAG CTC TCT CTC TCT CTT TGT TTG ACG CC-3' |
| DcF3'H-3-F | 5'-CCT GAC GTC GGT GCA ATA TTC GTC CTA AGA AAT TCA TG-3' |
| DcF3'H-4-R | 5'-CCT CTC GAG TGT CTC TCG GCC ATA TTG CCG AAC-3' |
| DcF3'5'H-3-F | 5'-CCT GAC GTC CCA ACT TTA TAG AAA GCC TAC AGC TCTATTTATAGC-3' |
| DcF3'5'H-4-R | 5'-CCT CTC GAG AGT GGA GTT TGT TAG CTT CAA GAG CCT TTC-3' |
| DcDFR1-3-F | 5'-CCT GAC GTC CCA ATT TCA GAC GGA CAT CTT AAA TCT C-3' |
| DcDFR1-4-R | 5'-CCT CTC GAG TTC AGT TCA ACT CAT GCA CAG CAC-3' |
| DcDFR2-3-F | 5'-CCT GAC GTC GAG TTT ACC TTC AAC TCA CTC TCT AGT CTT ATC-3' |
| DcDFR2-4-R | 5'-CCT CTC GAG GCC TTC TCA GCA AGT GTT TTT GAT AGG-3' |
| DcANS-3-F | 5'-CCT GAC GTC GGG AGA TCA AAA GAA GCA TGG CAA AG-3' |
| DcANS-4-R | 5'-CCT CTC GAG TGT GTA TTC TTA GTG AGT AGC TTT GGC TG-3' |
| DcUFGT-3-F | 5'- CCT GAC GTC TCA GTT AAT TGT AAA AGT AAG TAG TTT TTT TG-3' |
| DcUFGT-4-R | 5'-CCT CTC GAG GGC CAG AAA GCT TGA ACC AGA TGG AAG GGA TG-3' |

**Table S5** Sequencing data statistics.

| **Sample** | **Raw reads** | **Raw bases** | **GC (%)** | **N (%)** | **Q20 (%)** | **Q30 (%)** |
| --- | --- | --- | --- | --- | --- | --- |
| RDc1000-1 | 24689766 | 7.37G | 46.07 | 0 | 98.07 | 94.46 |
| RDc1000-2 | 22040860 | 6.58G | 46.8 | 0 | 97.99 | 94.28 |
| RDc1000-3 | 29123287 | 8.63G | 46.35 | 0 | 98.14 | 94.63 |
| RDc4000-1 | 22219664 | 6.63G | 46.61 | 0 | 98.22 | 94.72 |
| RDc4000-2 | 22605529 | 6.74G | 46.59 | 0 | 98.04 | 94.36 |
| RDc4000-3 | 25662403 | 7.67G | 46.4 | 0 | 98.11 | 94.45 |

**Table S6** Alignment results for each sample.

| **Sample** | **Total reads** | **Mapped reads** | **Unique map** | **Multiple map** | **Pair map** | **Single map** |
| --- | --- | --- | --- | --- | --- | --- |
| RDc1000-1 | 49379532 | 44686114  (90.5%) | 43332684  (87.75%) | 1353430  (2.74%) | 42456532  (85.98%) | 2229582  (4.52%) |
| RDc1000-2 | 44081720 | 40113993 (91.0%) | 38912120 (88.27%) | 1201873 (2.73%) | 38077608 (86.38%) | 2036385 (4.62%) |
| RDc1000-3 | 58246574 | 52762148 (90.58%) | 51375338 (88.20%) | 1386810 (2.38%) | 50644450 (86.95%) | 2117698 (3.64%) |
| RDc4000-1 | 44439328 | 40394056 (90.90%) | 39188505 (88.18%) | 1205551 (2.71%) | 38446206 (86.51%) | 1947850 (4.38%) |
| RDc4000-2 | 45211058 | 41151913 (91.02%) | 39912736 (88.28%) | 1239177 (2.74%) | 39164852 (86.63%) | 1987061 (4.40%) |
| RDc4000-3 | 51324806 | 46745319 (91.08%) | 45348878 (88.36%) | 1396441 (2.72%) | 44436262 (86.58%) | 2309057 (4.50%) |

**Table S7** Transcription factors among DEGs in RDc4000 vs. RDc1000 as determined by RNA-seq.

| **ID** | **log2 (FC)** | **Regulated** | **NR annotation** | **Transcription factor family** |
| --- | --- | --- | --- | --- |
| gene26293 | -1.11649 | down | ethylene-responsive transcription factor RAP2-1-like | AP2/ERF |
| gene18917 | -1.08737 | down | AP2/ERF and B3 domain-containing protein Os05g0549800-like AP2/ERF | AP2/ERF |
| gene19492 | -1.09503 | down | zinc finger protein CONSTANS-LIKE 16 | B-box zinc finger |
| gene11286 | -1.25035 | down | protein BZR1 homolog 1-like | BES1/BZR1 |
| gene5400 | -1.65244 | down | Transcription factor bHLH62 | bHLH |
| gene22107 | 3.890294 | up | Transcription factor TT8 | bHLH |
| newgene22313 | 1.943985 | up | transcription factor HY5-like | bZIP |
| gene16939 | -1.29912 | down | homeobox-leucine zipper protein HOX14-like | HD-Zip |
| gene9790 | -1.14575 | down | homeobox-leucine zipper protein HOX19-like | HD-Zip |
| gene14986 | -1.1845 | down | homeobox-leucine zipper protein HAT3-like | HD-Zip |
| gene3968 | -1.16236 | down | Agamous-like MADS-box protein AGL8 | MADS |
| gene11431 | 1.001346 | up | MADS-box transcription factor 50 | MADS |
| gene3059 | 1.124225 | up | Myb-related protein 308 | MYB |
| gene6015 | 3.149152 | up | trichome differentiation protein GL1-like | MYB |
| gene12101 | 1.273552 | up | NAC domain-containing protein 68-like | NAC |
| gene13414 | -1.23338 | down | NAC transcription factor 29 | NAC |
| gene24557 | -1.34277 | down | NAC transcription factor 29-like | NAC |
| gene2267 | -1.71257 | down | NAC domain-containing protein 100-like | NAC |
| gene21950 | -1.02197 | down | transcription factor PCF2-like | TCP |
| **gene11389** | **4.008653** | **up** | **protein TRANSPARENT TESTA GLABRA 1-like** | **WD40-repeat** |
| gene20726 | 2.041992 | up | probable WRKY transcription factor 34 | WRKY |
| gene7502 | -1.17891 | down | probable WRKY transcription factor 65 isoform X1 | WRKY |

**Table S8** Eight DEGs related to flavonoid biosynthesis pathways.

| ID | log2 (FC) | regulated | NR_annotation | KO id (EC no.) |
| --- | --- | --- | --- | --- |
| gene24598 | 4.46 | up | Naringenin,2-oxoglutarate 3-dioxygenase | K00475 (EC:1.14.11.7) |
| gene22054 | 4.72 | up | Dihydroflavonol-4-reductase | K13082 (EC:1.1.1.21) |
| gene16265 | 4.28 | up | Flavanone 3-dioxygenase | K00475 (EC:1.14.11.9) |
| gene12863 | 4.23 | up | Dihydroflavonol-4-reductase | K13082 (1.1.1.234) |
| gene14577 | 3.53 | up | Leucoanthocyanidin dioxygenase | K05277 (EC:1.14.11.19) |
| gene2797 | 2.08 | up | Chalcone synthase 8 | K00660 (EC:2.3.1.74) |
| gene5302 | 1.22 | up | Chalcone-flavonone isomerase | K01859 (EC:5.5.1.6) |
| gene19829 | 3.57 | up | Flavonoid 3'-monooxygenase CYP75B3-like | K05280 (EC:1.14.13.21) |


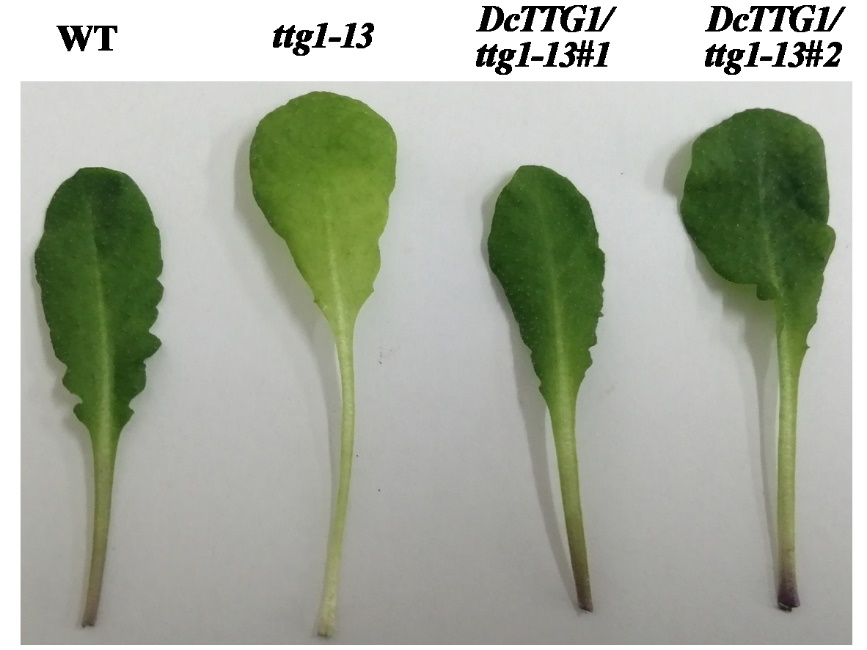


**Figure S1 Phenotypes of leaves** **in** **WT, *ttg1*, *DcTTG1/ttg1-13#1*, and *DcTTG1/ttg1-13#2*.** Overexpression of *35S:DcTTG1* in the *ttg1-13* background rescues the mutant phenotype of anthocyanin production.
